# Infants use helping to infer the existence and strength of caring relationships

**DOI:** 10.1073/pnas.2531771123

**Published:** 2026-04-10

**Authors:** Bill Pepe, Brandon M. Woo, Ashley J. Thomas, Lindsey J. Powell

**Affiliations:** ^a^Department of Psychology, University of California San Diego, La Jolla, CA 92093; ^b^Department of Psychological and Brain Sciences, University of California Santa Barbara, Santa Barbara, CA 93106; ^c^Department of Psychology, Harvard University, Cambridge, MA 02139

**Keywords:** prosocial, help, hinder, relationships, infant

## Abstract

Prosocial and antisocial behaviors can reflect the actor’s positive or negative feelings toward a specific person and their broader disposition toward others’ welfare. Observers’ inferences about these motives shape what they learn about the actor’s social relationships and moral character, thereby informing expectations about the actor’s future behaviors. In four preregistered studies, we used violation-of-expectation looking methods to investigate 14- and 15-mo-old infants’ inferences about social motives by first showing them helping and hindering events and then assessing their expectations for those actors’ future actions. If infants use helping and hindering to infer disposition-based motives, they should extend expectations about future helping to others. Alternatively, if they infer relationship-based motives, their expectations should be restricted to the initial interactants. In Experiments 1 and 2, infants first observed two actors, one who helped and another who hindered a common target. At test, infants expected the helper would help the same target in a new, perceptually distinct situation. However, this expectation did not generalize to a new target, suggesting infants inferred specific relationships, not general dispositions. In Experiments 3 and 4, we assessed whether infants used information about the existence or strength of a helper’s relationships to predict who they would prioritize helping in the future. We found that infants used patterns of selective helping among multiple targets to make transitive inferences about whose needs the helper would prioritize. Taken together, this suggests that infants use such observations of helping to infer whether, and how much, the helper cares for their social partner.

## Significance

Decades of work suggest that human infants distinguish prosocial and antisocial behavior. However, less is known about what infants infer from such behaviors. People can behave prosocially for at least two different reasons: Their actions may reflect a disposition, or they may reflect a relationship with their interaction partner. By inferring the reason that a person helps or hinders others, an observer can gain insight into how the person will act in the future. Here, we found evidence that infants interpret helping and hindering actions as evidence for the existence and strength of the actor’s relationship with the recipient, rather than the actor’s individual disposition. Our results support the foundational role of relationship representations in early social reasoning.

Social beliefs profoundly shape one’s experience of the world: Do my family and friends care about me (1–3)? Are the people around me generally kind and trustworthy (4–7)? How am I expected to treat others (8–11)? The answers to these questions depend not just on the behaviors a person observes but also on the inferred motives for those behaviors. Observers may see an act of helping as motivated by altruistic moral values, by social obligation, by parochial bias, or by the goal of eliciting a reciprocal favor (12–18), and each of these inferred motives would lead to different conclusions about the helper. While the full breadth and flexibility of this reasoning takes years to develop (9, 19–22), the basic tendency to see social behaviors as reflective of underlying motives may develop much earlier. If so, infants’ developing beliefs about social motives would shape what they learn from observing others’ interactions.

**Fig. 1.**
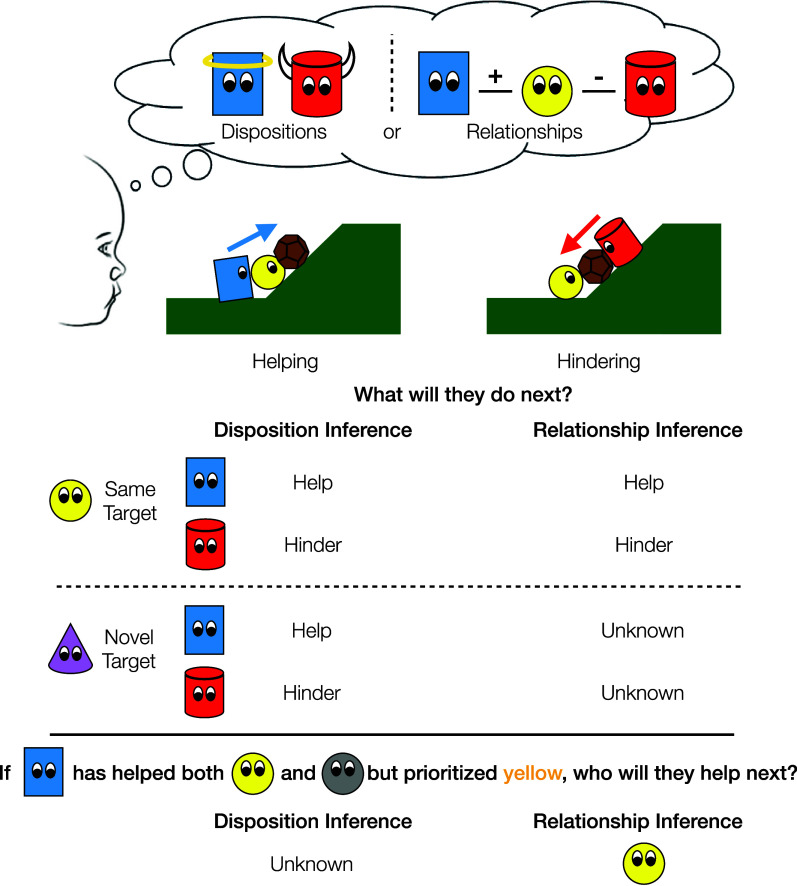
Overview of hypotheses. When infants observe helping and hindering actions, they could infer that the actors have prosocial and antisocial dispositions, respectively, or they could make inferences about the actors’ relationships to the target. These alternatives both predict that the observer will expect the actors to continue to help and hinder the same target, but only a disposition inference predicts similar expectations of helping and hindering toward a new target. In a different scenario, an observer may see an actor help multiple social targets over a series of events, while consistently favoring some targets over others. Inferring the helper’s prosocial disposition alone would not allow the observer to predict who the helper will help next, but inferring the strength of the helper’s relationship to each target would support such specific predictions.

In four preregistered experiments, we investigated 14- and 15-mo-old infants’ inferences about the motives underlying social actions by asking about their expectations for future behavior. Specifically, we ask if infants interpret helping and hindering as evidence of general social dispositions or, alternatively, positive or negative social relationships between observed actor-target pairs. Although these inferences are not mutually exclusive, they predict different patterns of expectations. If infants use observations of helping and hindering to make inferences about the actors’ dispositions—i.e., their general tendency to promote or diminish others’ welfare—then infants should expect these actors to engage in similar helping or hindering behavior in future situations, regardless of whether the target is the same or a new individual. Alternatively, if infants use these social observations primarily to make inferences about the relationships between actors and the recipients of their actions, then their expectations about the actors’ future behavior may be limited to situations in which they interact with the same social partners. Inferring the strength of different relationships would also allow infants to predict which social partner a helper would prioritize, in a way that inferring the strength of a prosocial or antisocial disposition would not (Fig. 1).

The largest body of work on infants’ understanding of prosocial and antisocial behavior has focused on infants’ evaluations of helpers and hinderers. Studies that find an early-developing preference to reach for or look at helpers over hinderers have often concluded that the participating infants infer the helpers have morally good dispositions (23–26). However, these studies do not test this claim directly, and relationship-based motives may provide an alternative basis for evaluation: Infants, similar to adults and older children, may value potential relationship partners who display reliability or loyalty (18, 27–29). There may also be cases in which infants perceive helpers’ or hinderers’ relationships to their targets as self-relevant, prompting positive or negative responses (30, 31). A preference for people who direct prosocial behaviors and traits toward oneself and one’s social partners would be consistent with evidence that adults’ trait-based evaluations are significantly influenced by their own relationships to the actors and targets (32, 33). When adult participants are asked to evaluate a person based on how they treat the participant’s rivals or outgroup, adults’ typical preference for prosocial traits can reverse, such that they positively evaluate people who act in antisocial ways toward these targets (32, 33). In sum, infants’ preferences do not offer clear information about their underlying inferences about motives. Instead, this requires directly investigating infants’ expectations about helpers’ future behavior, and the consistency of those expectations with either relationship or disposition inferences.

While prior work has not directly tested for these hypothesized patterns, there is some relevant evidence. Duh et al. (34) found that infants dishabituated when an actor switched from helping to hindering or vice versa, with all events featuring the same recipient. This could reflect inferences about either the actor’s disposition or relationship to the recipient, or it could indicate general encoding of helping and hindering event types. In addition, Taborda-Osario et al. (35) found that infants used the helping and hindering actions of two identical-looking agents, each appearing alone in different trials, to infer they were separate individuals both hidden behind the same occluder. While the authors hypothesized infants’ individuation was based on attribution of two different social dispositions (i.e., one “nice” agent and one “mean” one), it is also possible that individuation was based on the inference of agents’ different relationships, one positive and one negative, to the common target of their prosocial and antisocial actions.

Several other studies have tested for moral disposition inferences in infants and toddlers between 14 and 30 mo by asking if observations of helping or hindering affect infants’ expectations about other types of social behaviors, such as fairness in resource distribution directed toward new targets (36–38). Whereas infants usually expect people to distribute resources equally, these studies generally find that infants suspend this expectation for hinderers. It is difficult to clearly interpret this finding as evidence of a disposition inference, however, as infants also did not expect the hinderer to be unfair, nor did they tend to show concurrent expectations of both helping and fairness from the same actor. Thus, it remains unclear whether infants attribute prosocial or antisocial dispositions to agents who help or hinder others.

Other studies have tested infants’ expectations following other prosocial or intimate behaviors, such as giving, imitating, or sharing food and saliva, and found evidence consistent with relationship inferences (39–43). Infants expect the prosocial actor in these scenarios to engage in further positive, supportive, or intimate actions (e.g., helping or comforting) toward the same social partner, but not a new one. However, particular kinds of interactions may elicit different inferences about underlying causes, with some social behaviors selectively associated with relationships, dispositions, or both (43, 44). Thus, infants’ expectations following helping and hindering may or may not follow a similar relationship-specific pattern.

We tested these expectations directly in four preregistered “violation of expectation” (45, 46) experiments with 14- and 15-mo-old infants (N = 52 per experiment; for preregistrations, materials, and data, see OSF Repository (47)). We asked if infants 1) expect a helper to continue to help the same social partner only or also a new social partner (Experiments 1 and 2), and 2) if they could use a helper’s previous choices about who to help to predict whose needs the helper would prioritize in a new situation (Experiments 3 and 4). Together, the findings from these experiments test whether infants use helping and hindering to recognize the existence and strength of one agent’s care for another’s welfare, and if they do so by inferring the agent’s broad disposition to help or their relationship to a specific social partner. We selected an age range early in the second year of life because infants this age understand how to help others in a variety of situations and also show expectations about others’ social behavior, raising the likelihood that they infer underlying social motives for acts of helping and hindering.

## Results

### What Will Helpers and Hinderers Do Next?

#### Experiment 1.

Before testing which cause—dispositions or relationships—infants use to explain helping and hindering, we first tested whether infants’ expectations about others’ likelihood of helping are guided by their past behavior at all. To do this, Experiment 1 asked if infants would expect a past helper to be more likely than a past hinderer to help the same target in a new situation with different surface features and goals. Since both helping and hindering actions were presented during familiarization such expectations could not reflect mere habituation to an event type.

**Fig. 2.**
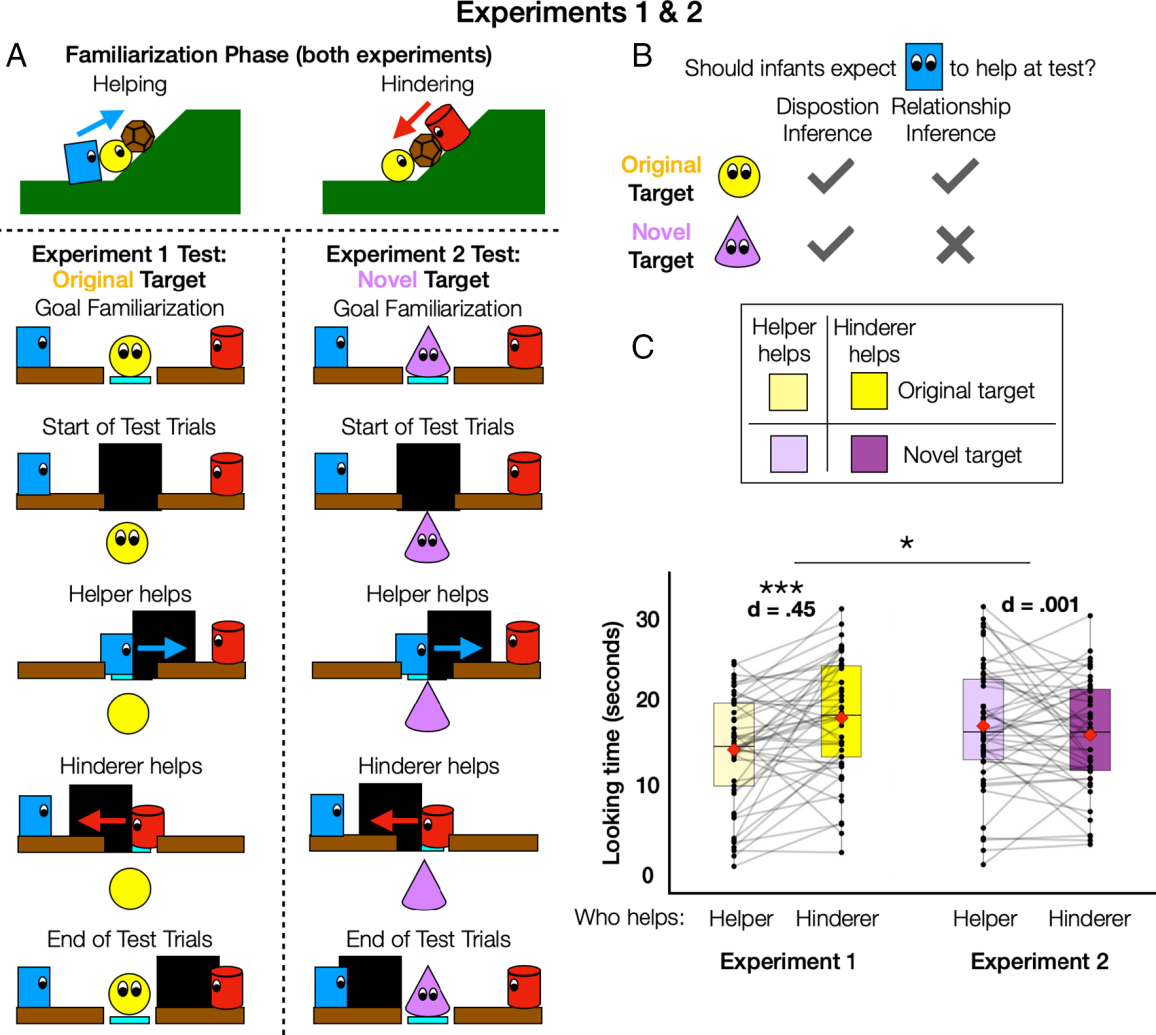
Experiment 1 and 2 materials and results. (*A*) Schematic illustrations of the events shown in Experiments 1 and 2. Both experiments began with a familiarization phase in which one actor helped a target character push a boulder up a hill and another actor pushed the target and boulder back down the hill. In the test phase of Experiment 1, the helper and hinderer each helped the same target get past a large wall to a goal location; in Experiment 2, the helper and hinderer each helped a novel target in the same way. (*B*) Diagram of the logic linking the experiments to competing hypotheses about infants’ use of the behaviors in familiarization to infer the helper’s prosocial disposition or positive relationship to the original target. (*C*) Figure legend and results of Experiments 1 and 2. Mean looking time in seconds (before log-transform) toward each trial type from each experiment. Black points indicate participant-level data, with gray lines connecting data from the same participant. Red diamonds indicate group mean.

First, infants saw six total familiarization events, alternating between helping and hindering. In each event, a yellow, spherical target character struggled to push a boulder up a hill (23, 48). One of two other characters then intervened, either by helping to push the character and boulder up the hill or by blocking the target, pushing it and the boulder back down the hill (Fig. 2*A*).

The same characters then appeared in a new scene, and participants saw a goal familiarization event, in which the target character demonstrated a new goal to reach a platform via a path between two low walls. The helper and hinderer were located at opposite ends of the low walls and watched the target’s movements (Fig. 2*A*).

Infants then saw four test trials in which a larger wall blocked the path leading to the platform. In alternating events, the helper or hinderer moved the wall out of the target’s path, allowing the target access to their goal. We hypothesized that infants would look longer, indicating surprise, when the hinderer helped by moving the wall, compared to events in which the helper continued to help (Fig. 2*B*).

A nested comparison of mixed effect models (*Materials and Methods*) revealed a main effect of trial type on looking time, with infants (N = 52, 14 to 15 mo) looking significantly longer to the test trials in which the hinderer helped (raw *M* = 20.9 s, SD = 8.64 s), than to trials in which the helper helped (raw *M* = 17.2 s, SD = 8.87 s), X^2^ = 11.95, *P* < 0.001, d = 0.45 (Fig. 2*C*). This suggests that infants expected the helper, rather than the hinderer, to continue helping when the target needed assistance in a new situation. (For complete results of this and other experiments, see *SI Appendix*).

#### Experiment 2.

We next tested if infants’ expectations generalize to a novel social target. If so, this would suggest that infants infer helpful and hindering dispositions or roles, while a failure to extend their expectations to new targets would suggest relationship inferences (Fig. 2*B*). Displays were the same as in Experiment 1, except that the yellow target agent was replaced by a new target agent in the goal familiarization and test trials (Fig. 2*A*). The novel target, a lavender-colored, cone-shaped character, differed from the original target in both color and shape to ensure infants could differentiate between the two targets.

Data from Experiment 2 (N = 52, 14 to 15 mo) revealed no main effect of trial type, as infants’ looking time did not differ significantly between test trials in which the helper (raw *M* = 19.6 s, SD = 8.81 s) or the hinderer (raw *M* = 19.4, SD = 9.08 s) helped the novel target, X^2^ = 0.01, *P* = 0.89, d = 0.001 (Fig. 2*C*). Experiment 2 thus did not provide evidence that infants’ expectations of continued helping and hindering extended to the novel target. A preregistered comparison of the data from Experiments 1 and 2 revealed a significant interaction between trial type and help recipient, X^2^ = 4.77, *P* < 0.05, η^2^= 0.11, indicating that infants’ lack of expectation about who would help the novel target differed reliably from their expectation that the helper would continue to help the original target.

Together, data from Experiments 1 and 2 are consistent with the hypothesis that infants use actors’ past helping and hindering to guide expectations of future behavior, and that these expectations are based on inferences about stable support for a particular relationship partner, rather than general social dispositions. However, it is also possible that infants’ expectations in Experiment 1 were the product of disposition inferences, and that the null result in Experiment 2 reflects the fragility of these inferences. For instance, infants may struggle to maintain and use disposition inferences when the identity or number of characters change between scenes. The conclusion that infants can and do use basic observations of helping and hindering to infer relationships would be better supported by positive evidence for expectations about helpers’ or hinderers’ future behavior that would uniquely follow from inferences of relationships but not dispositions. In the next two experiments, we thus sought to provide positive evidence that infants can make and then maintain relationship-based inferences across scenes with changing sets of characters.

### Do Infants Infer Strength of Care?

People have numerous, distinct relationships that are situated within a broader social network. People do not, however, have infinite time or resources. Thus, an important aspect of reasoning about relationships involves predicting which social partner(s) a person will prioritize in different situations. Children and adults can base such predictions on the kinds of relationships that people have: They believe people have different obligations to friends and family than to acquaintances or strangers (18, 48–52). However, people also recognize continuous gradations in strength of care within and across relationship categories (44, 52–56), which can inform expectations about how much a person will sacrifice in order to help and which social partner will be prioritized when not all can be aided at the same time (49, 52, 57). In Experiments 3 and 4 we investigated infants’ ability to use both the existence and the strength of caring relationships to figure out who will receive help.

#### Experiment 3.

In Experiment 3, we investigated whether infants expect helpers to prioritize the needs of their previous target over a new target. Participants in Experiment 3 saw similar familiarization events to Experiments 1 and 2, with a new set of characters. One actor helped, and another hindered the same target character. At test, however, both that original target and a new target were present, and the hinderer was gone. We tested who infants expected the helper to help: the original target or the new target?

**Fig. 3.**
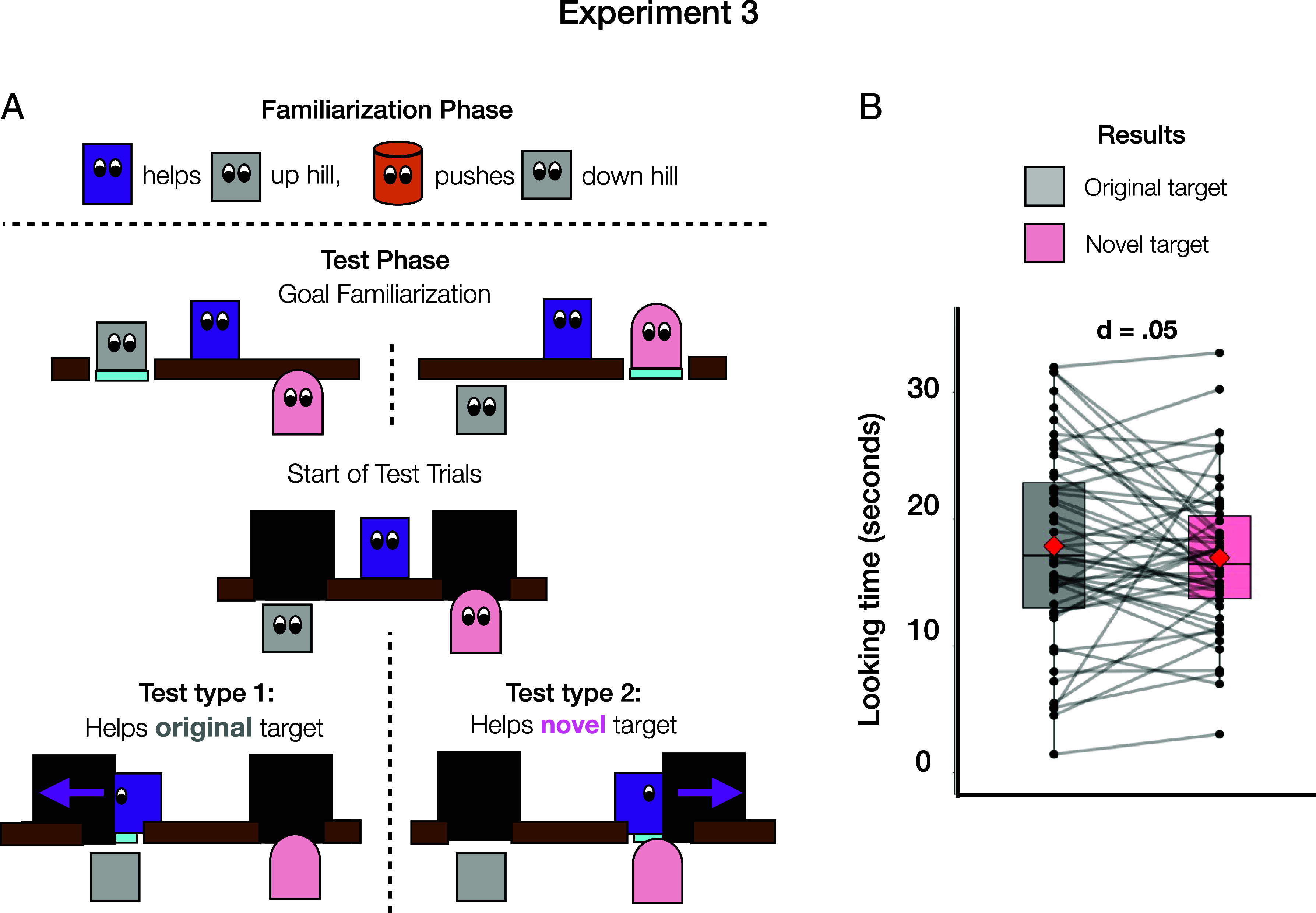
Experiment 3 materials and results. (*A*) Schematic illustrations of the events shown in Experiment 3. In the familiarization phase one actor helped a target character push a boulder up a hill and another actor pushed the target and boulder back down the hill. In the test phase, the helper helped either the same target or a novel target get past a large wall to a goal location. (*B*) Figure legend and results of Experiment 3. Mean looking time in seconds (before log-transform) toward each trial type. Black points indicate participant-level data, with gray lines connecting data from the same participant. Red diamonds indicate group mean. Outlying datapoints are beyond ±1.5 SD from the upper and lower quartiles.

In displays for the goal familiarization event and test trials, the platform-and-path setup from the test trials of Experiments 1 and 2 was duplicated on each side of the screen. The helper was positioned in the middle, on a low wall separating the two paths (Fig. 3*A*). In the goal familiarization event, the original and novel targets took turns going to the platform located on their side of the display. In the test trials, two large walls blocked each target’s path to their goal platform. The helper then chose to help either the original or novel target by moving the wall out of their path and then returned to their starting location.

We reasoned that a relationship inference made during the initial familiarization events would lead infants to expect the helper to help their original target, looking longer when the helper chose the novel target. However, this is not the only plausible hypothesis involving relationship inferences: If infants consider that the helper could care more for the novel target than the original one, then they may not have strong expectations and may be interested in the novel information about relative strength of care for the two targets provided by either trial type. Experiment 4 directly addresses this possibility. (A secondary goal of Experiment 3 was to test for individual differences in infants’ tendency to infer relationship vs. disposition motives. To this end, we recruited participants from Experiment 2 to participate in Experiment 3, and the samples were largely overlapping. See *Materials and Methods* for more on the sample overlap and *SI Appendix* for analyses and results.)

A preregistered nested comparison of mixed effect models did not show a reliable difference in looking time to test trials in which infants (N = 52, 14 to 15 mo) saw the helper assist the novel target (raw M = 18.0 s, SD = 8.22 s) vs. the original target (raw M = 18.3 s, SD = 8.22 s), X^2^ = 0.08, *P* = 0.78, d = 0.05 (Fig. 3*B*). We thus did not find evidence that infants expected the helper to selectively help their previous target. There is a range of potential explanations for this null result, but one possibility is that infants were interested in novel information about the helper’s relative strength of care for each potential target, which was provided by both trial types.

#### Experiment 4.

**Fig. 4.**
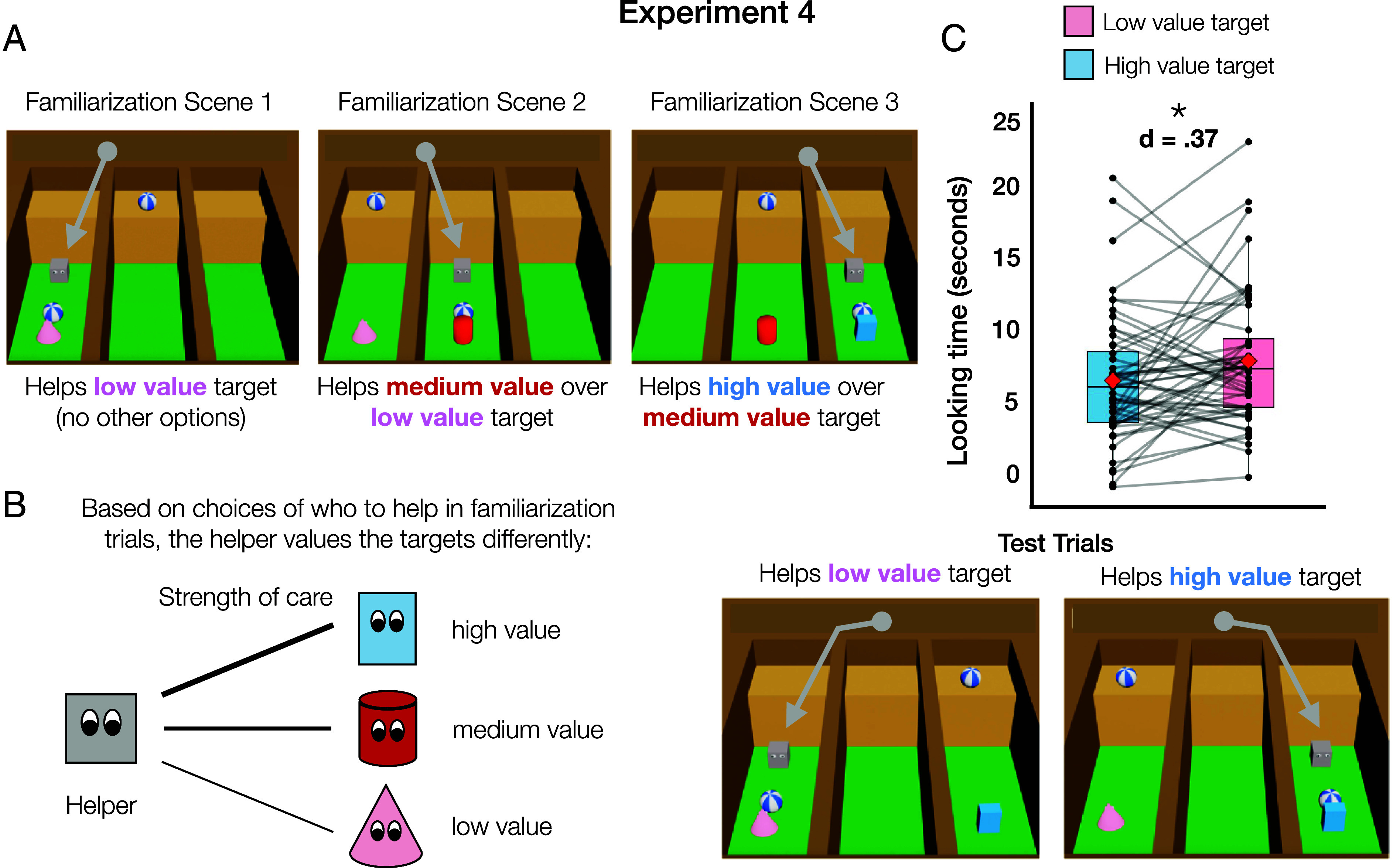
Experiment 4 materials and results. (*A*) Examples of the displays used in Experiment 4. In the familiarization phase, each scene depicted one or two characters who needed help reaching a goal object. The helper, initially located on the high plateau toward the back of the display, picked one target and descended down into their alley to help them get the object. The helper consistently prioritized the same targets as depicted. In the test phase, the helper had a new choice of targets and alternated between helping each of them. (*B*) Diagram of potential inferences about how much the helper cares about the welfare of each target, given its prioritization of their welfare during familiarization. (*C*) Figure legend and results of Experiment 4. Mean looking time in seconds (before log-transform) toward each trial type. Black points indicate participant-level data, with gray lines connecting data from the same participant. Red diamonds indicate group mean. Outlying datapoints are beyond ±1.5 SD from the upper and lower quartiles.

We tested this explanation for the null results in Experiment 3 by asking whether infants use a helper’s choices about who to help to infer how much the helper cares about different social partners. To do this, we tested for transitive inferences that would depend on infants using a helper’s choices to map their care for each potential target onto a common underlying scale. Following work on infants’ transitive inferences about object preferences and about social dominance (58, 59), we showed infants a helper who helped three recipients (i.e., targets “A,” “B,” and “C”) an equal number of times, but who prioritized helping B over A and C over B (Fig. 4*A*). We then showed them test trials in which the helper had to choose between helping A and C, a previously unseen target pairing, and used looking time as a measure of who infants expected the helper to prioritize.

We created a new type of helping event to implement this study (Fig. 4*A*). In each trial, the helper was on a plateau overlooking a series of three alleys, which could each be occupied by a specific target character. One or two target character(s) were present at a time, and each attempted, but failed, to reach a ball that had rolled onto a shelf in their alley, near the helper’s plateau. The helper decided who to help by dropping onto one of the shelves and pushing the ball to the target, who then celebrated.

Across nine familiarization events, infants saw three event types three times each. The helper 1) helped A when only A was present, 2) helped B over A, or 3) helped C over B (Fig. 4*A*). The event sequence consisted of three repetitions of these events in either A-B-C or C-B-A order, counterbalanced across participants. At test, targets A and C were present together and infants saw the helper alternate between choosing to help target “A” (i.e., low-value target) and target “C” (i.e., “high-value” target). If infants understand a helper’s choices reflect their strength of care on a continuous scale (Fig. 4*B*), then they should expect the helper to help C rather than A. If they do not track strength of care in this way, then they may be at chance, particularly given that they saw the helper assist A and C an equal number of times during familiarization.

A nested comparison of mixed effect models revealed a main effect of trial type on infant looking time, with infants (N = 52, 14 to 15 mo) looking significantly longer when the helper helped the low-value target (raw M = 8.74, SD = 4.58), than to trials in which they helped the high-value target (raw M = 7.39 s, SD = 4.41 s), X^2^ = 5.58, *P* < 0.05, d = 0.37 (Fig. 4*C*). This suggests infants were able to use the helper’s choices during familiarization to infer the relative strength of their care for each target, which then guided their expectations about who the helper would help when presented with a novel choice between recipients.

The findings from Experiment 4 provide further support for the conclusion that infants infer relationship-based motives for acts of helping. Infants’ systematic expectations for whose welfare the helper would prioritize could not arise from inferences about the helper’s disposition alone. Instead, these expectations required infants to represent the relative strength of the helper’s commitment to or care for each social partner using a common underlying scale, allowing them to predict who would receive help in a new situation.

In addition, Experiment 4 addresses one potential explanation for the null results of Experiment 2, in which infants did not expect the helper’s actions to generalize to a new target: Experiment 4 shows that this cannot be the result of a general inability to maintain expectations across changes in the set of agents involved in different events. This increases our confidence that Experiment 2 reflected a true failure to attribute prosocial and antisocial dispositions to the helper and hinderer, though future research should address the remaining possibility that co-occurring changes in both target and goal affect the robustness of infant’s expectations.

## Discussion

Across four preregistered experiments, we assessed whether infants use observations of helping and hindering to infer social motives that guide expectations about future behavior. In Experiments 1 and 2 we found evidence that infants expect helpers and hinderers to behave similarly in a new context, but only when their actions are directed toward the same recipient. This suggests that infants used their observations to infer stable, specific relationships between the two actors and the target of their prosocial or antisocial actions. In Experiment 3, we found that such relationship inferences do not necessarily lead infants to expect that a helper will prioritize a previous social partner over a new potential target. This is consistent with the possibility that infants are interested in learning about the strength of helpers’ care for different relationship partners. The results of Experiment 4 supported this hypothesis: Infants were able to use patterns of selective helping to map the helper’s relative strength of care across social partners, supporting transitive inferences about which partner the helper would help in a new situation. The results from Experiment 4 also suggest that infants can learn and maintain information about a helper’s motives even as the recipient of their help varies, indicating that the differences found between Experiment 1 and 2 could not be attributed to changing sets of characters across events.

These findings support the conclusion that infants infer social relationships as motives for helpful behavior. They do not provide any evidence that infants also infer prosocial or antisocial dispositions on the basis of helping or hindering. The findings also cannot be explained by associations between agents and roles or event types (60), and it is unlikely that low-level motion dynamics can explain the current results (61). The helper and hinderer both approached the target in the familiarization events of Experiment 1, one then moving congruently and one incongruently with the target’s intended motion path. In contrast, helping during the test trials involved approaching a wall, rather than the target agent, and moving along a path perpendicular to the target’s path.

These experiments leave open questions about the format of infant relationship representations. The results are consistent with the proposal that infants represent how much one individual values another’s welfare, which could be one feature of a multidimensional relationship representation (62, 63). When integrated with a broader framework of intuitive psychology that connects what an actor values with their beliefs, actions, and emotions, representations of interpersonal valuation, or “care,” can support forward and backward inferences about how care shapes social interactions and interpersonal emotions, like the inferences demonstrated here and in other recent research (34, 39, 40, 49, 62, 64–67). The results are also compatible with other, more domain-specific accounts of early relationship representations, which propose that infants do not compute costs and benefits when recognizing or reasoning about relationships (44).

The current findings inform our understanding of infants’ social evaluations. They show that infants do form impressions of helpers that support systematic expectations of future helping. Together with the emergence of competent helping behavior in the second year of life (68–71), this suggests that failures to prefer helpers to hinderers in this same age range (72) do not reflect an inability to understand helpful actions. However, these findings do suggest we should question the view that infants’ preference for helpers, in basic social interactions, similar to those depicted in the current experiments, is based on a dispositional inference of helpers’ moral goodness (23, 24, 26, 73) and consider alternative, relationship-based accounts (44, 62). Such relationship-based accounts would be consistent with how adults use their understanding of people’s social relationships when evaluating the moral valence of others’ actions (11, 32, 33).

Our results are at odds with past claims that infants infer antisocial dispositions from hindering in displays similar to ours (36–38). While infants usually look longer at unequal distributions, they look equally long at equal and unequal distributions when done by hinderers. One possibility is that infants do, in fact, infer antisocial dispositions from hindering, but tend to infer relationships from helping. This would seem to predict, however, that infants should look longer when a previous hinderer helps, then when a helper helps, a new social target, which is inconsistent with our findings from Experiment 2. An alternative possibility is that inferences about relationships may have played a role in the previous research the assessed infants’ expectations of equal distributions. For instance, infants may typically assume that someone distributing resources is a leader or caregiver, leading them to expect equal distribution. They may see someone with antisocial relationships as unlikely to occupy such a role, altering their expectations in these resource allocation situations. This possibility could be explored in future research.

Future research should also investigate whether different input would lead infants to attribute prosocial dispositions. Adults and older children use the consistency of social behaviors across time, setting, and social partners to infer dispositions or traits (74–77). In Experiments 1 and 4 infants saw either narrow or biased patterns of helping, so their relationship inferences may thus reflect a rational interpretation of the evidence rather than a limit on the social inferences they can make (78–80). Additional studies should test whether familiarization with egalitarian helping, across more familiarization events and/or toward multiple targets would prompt infants to attribute prosocial dispositions and expect prosociality toward all social partners. Other factors that may spur infants to infer dispositions could include the costliness of helping or hindering, the degree of benefit or harm they produce, or the involvement of other social actions beyond helping and hindering (48). For example, fairness (and unfairness) may be more closely associated with disposition-based than relationship-based motives, given that it involves avoiding favoritism toward specific individual(s) (36). Inflicting harm, rather than merely blocking a goal, could be more likely to elicit the attribution of an antisocial disposition that would make the perpetrator’s future help toward any target individual surprising. We also did not test whether infants in Experiments 1 or 2 had expectations about future hindering behavior. A negativity bias (81, 82) could lead infants to find it most surprising to witness “nice” agents act in antisocial ways in the future, even toward new targets (c.f. 40, 83).

Should future research find evidence that infants infer dispositions in some contexts, it should also revisit questions about the interplay between disposition and relationship inferences. As noted above, we do not see them as mutually exclusive. In some cases, people may perceive them as correlated. For example, observers may think a person with many friends is likely to be kind and prosocial in general. Nonetheless, preferences for simplicity in causal explanations can sometimes lead to confidence in one type of motive to reduce attributions of the other (84). Thus, prosocial behaviors attributed to the actor’s kindness may be taken as only weak evidence for a positive relationship or vice versa, akin to cases in which the presence of self-serving benefits for helpful actions leads observers to doubt the simultaneous role of altruistic motives (85).

In sum, these findings support the hypothesis that infants can use observations of helping to reason about specific social relationships in their environment. This is consistent with recent work suggesting that infants use inferences about underlying relationships to interpret and connect a broad range of social behaviors (31, 39–42). Infants’ ability to use a helper’s choices to infer strength of care also complements findings that infants infer stronger or more intimate relationships from some types of social interactions than from others (43). Together, these findings support the idea that infants possess an abstract concept of social affiliation that allows them to organize observations and expectations about a range of prosocial behaviors between relationship partners (44, 62). This abstract knowledge of relationships offers a foundation for infants’ learning about their own social worlds.

## Materials and Methods

### General Procedure and Design.

The sample size of each experiment provided 80% power to detect medium-sized effects of expected vs. unexpected trial types on looking of d = 0.40 or higher, while allowing even counterbalancing (power analyses were carried out with G*Power) (86). Recruitment methods and study protocol were approved by the university review board at the University of California, San Diego (IRB#201677). Caregivers provided informed consent and were compensated with a $5 Amazon gift card. All infants were full term (gestation ≥ 36 wk). The sample sizes, procedures, experimental displays, hypotheses, and analysis plans were preregistered prior to data collection for each experiment, see OSF Repository.

All infants saw an initial calibration phase, a multicolored object with sound effects attracted infants’ attention to the corners of the screen to help guide coding. All experimental stimuli included animated, 3D-rendered geometric characters with eyes, created in Blender (87). Participants were tested remotely over Zoom using protocols demonstrated to elicit known looking time effects (88). Infants were seated in their parent’s lap or a highchair. Parents were instructed to not engage with their baby during the study (*SI Appendix*) and researchers monitored for compliance.

#### Coding and analyses.

For all infant-controlled trials, the online coder used Jhab (89) to code looking times as soon as the helping or hindering action began, cued by a relevant sound effect. The coder, who was naïve to trial type, initiated the next event once the infant looked away from the screen for 2 consecutive seconds or after 60 s of elapsed time. We did not code looking times during the goal familiarization events, which ran for a fixed duration, but repeated the event if participants did not see the target(s) jump onto the platform(s) at least once.

Test trials were coded frame-by-frame after the experiment, in Datavyu (90), by a coder naive to trial type. All exclusion criteria were preregistered. Test trials were excluded if infants looked away during the critical portion of the display depicting helping or hindering, though if the online coder noticed this occurred they replayed the trial for the infant. Trials were also excluded for parental or environmental interference, fussiness, inattentiveness, or issues caused by poor internet connectivity. If an individual trial was excluded for these reasons, we also excluded the paired trial of the other type, to minimize differential impact of strong trial order effects on looking times. If the remaining pair of test trials was unaffected, we retained the participant in the final dataset. If a participant did not contribute at least one valid pair of test trials, then their overall dataset was excluded and replaced. To assess coder reliability, 50% of the trials were recoded by an additional naive coder using Datavyu. Intercoder reliability was high, as determined by intraclass coder coefficients (ICCs) = 0.98-0.99, all *P*s < 0.001, with raw percent agreement between the two coders between 96.53% and 98.11% (*SI Appendix*).

Analyses were conducted on log-transformed looking times to correct for possible skew (91). We preregistered nested comparisons of mixed effects models to test for the impact of factors of interest on looking time in each experiment. The full models predicted log looking time to each trial as a function of the fixed factors of trial type and trial number, as well as the two-way interaction between these factors. Participant ID was included as a random effect, specified as intercept only. This full model was compared to reduced models that held out only one factor (e.g., the simple effect of trial type, but not the interaction of this effect with trial number) at a time.

### Experiment 1.

#### Participants.

Fifty-two 14- and 15-mo-old infants (M = 14.85 mo; SD = 0.48 mo; 22 females). Six pairs of test trials from this sample were excluded for parental or sibling interference (4), fussiness (1), and video lag (1). Four additional infants were excluded for fussiness (2) and video lag (2).

Thirty-two infants were identified by their caregiver as White, 3 as Asian or Asian American, 2 as Black, 11 as belonging to two or more races, and 4 caregivers chose not to respond. Eleven infants were identified by their caregiver as Hispanic/Latinx. Forty-six infants came from families in which at least one caregiver indicated having a college degree or higher.

#### Stimuli.

Infants saw helping and hindering familiarization events (three each), one goal familiarization event, and four test events. All events featured the same three characters, a spherical, yellow agent who acted as the “target” of helping and hindering, as well as a rectangular, blue character and a cylindrical, red character. See Fig. 2 for a schematic depiction of the events in this experiment and Experiment 2, and the project repository (OSF Repository) to view videos of the stimuli.

Which character appeared first during familiarization (Helper or Hinderer), the identity of the agents who helped and hindered during familiarization (Cylinder or Rectangle), and the order of helping actions at test (Unexpected or Expected first) were all counterbalanced. The rectangular and cylinder agents were always on the right and left, respectively, throughout familiarization and test; the side of the hill in familiarization trials varied across participants to facilitate counterbalancing roles.

### Experiment 2.

#### Participants.

Fifty-two 14- and 15-mo-old infants (M = 14.87 mo; SD = 0.51 mo; 27 females). Seven pairs of test trials from this sample were excluded for parental or sibling interference (3), fussiness (3), and inattention during critical test events (1). Three additional infants were excluded for fussiness (1) and video lag (2).

Thirty-four infants were identified by their caregiver as White, 5 as Asian or Asian American, 1 as Black, 11 as belonging to two or more races, and 1 caregiver chose not to respond. Thirteen infants were identified by their caregiver as Hispanic/Latinx. Fifty infants came from families in which at least one caregiver indicated having a college degree or higher.

#### Stimuli, coding, and analyses.

All stimuli and counterbalancing were identical to Experiment 1, except a novel target (lavender, cone-shaped character) replaced the yellow, spherical character during the goal familiarization and test trials.

In addition to the within-experiment analyses, we also preregistered a cumulative analysis combining the data from Experiments 1 and 2. This analysis augmented the full model with the fixed effect of target identity (original target/Exp 1 vs. novel target/Exp 2) and its two- and three-way interactions with the factors of trial type and trial number. We again conducted nested model comparisons by omitting a single simple or interaction effect at a time. We hypothesized that if the target’s identity impacted infants’ expectations about which of the two social actors would help, then the full model would explain significantly more variance than a reduced model omitting the interaction between target identity and trial type.

### Experiment 3.

#### Participants.

Fifty-two 14- and 15-mo-old infants (M = 15.32 mo; SD = 0.66 mo; 28 females). Families of infants who participated in Experiment 2 were asked to also participate in Experiment 3 within 30 d of their first appointment (see *SI Appendix* for the hypotheses, methods, and results of a planned investigation of individual differences). Forty-nine families did so; three additional participants were recruited to replace the infants who did not return for a second appointment. Two pairs of test trials (and no full datasets) were excluded for parental or sibling interference (1) and fussiness (1).

Thirty-four infants were identified by their caregiver as White, 5 as Asian or Asian American, 1 as Black, 11 as belonging to two or more races, and 1 caregiver chose not to respond. Eleven infants were identified by their caregiver as Hispanic/Latinx. Fifty infants came from families in which at least one caregiver indicated having a college degree or higher.

#### Stimuli.

The helping/hindering familiarization events were identical to Experiments 1 and 2, except that a different set of characters appeared in Experiment 3, including a gray cube which acted as a target character in both familiarization and test trials, a dark purple rectangle and an orange cylinder that helped or hindered, and a pink, rounded character that acted as an additional, novel target in the test phase. The alternative set of characters was used to avoid infants who participated in both Experiments 2 and 3 remembering how the characters acted during the previous session. See Fig. 3 for a schematic depiction of the events in this experiment and the project repository (OSF Repository) to view videos of the stimuli.

The counterbalanced factors of Experiment 3 were identical to the previous experiments. The order of the goal familiarization events of Experiment 3 was consistent with the order in which the characters were helped during the test trials (i.e., unexpected/novel target helped first vs. expected/original target helped first).

### Experiment 4.

#### Participants.

Fifty-two 14- and 15-mo-old infants (M = 14.75 mo; SD = 0.50 mo; 27 females). Seven pairs of test trials from this sample were excluded for parental or sibling interference (1), fussiness (1), inattentiveness (4), and inattention to critical test events (1). Three additional infants were excluded for fussiness (1), parent interference (1), and video lag (1).

Thirty-two infants were identified by their caregiver as White, 8 as Asian or Asian American, 3 as Black, 8 as belonging to two or more races, and 1 caregiver chose not to respond. Thirteen infants were identified by their caregiver as Hispanic/Latinx. Forty-nine infants came from families in which at least one caregiver indicated having a college degree or higher.

#### Stimuli.

All events occurred in an identical setting, involving the same helper character and a subset of the same three target characters. Each event included one or two target characters, who needed help to obtain a ball that was out of their reach, and the helper, who decided which character to help in each trial. Each scene began with the helper positioned on top of a brown plateau along the back of the scene, which overlooked three alleys that were separated by brown walls. In the back end of each alley, close to the helper’s plateau, there was a yellow shelf. The ground of each alley was solid green. See Fig. 4 for example scenes from this experiment and the project repository (OSF Repository) to view videos of the stimuli.

The following factors were all counterbalanced: Which character was helped first during familiarization (low-value target or high-value target), which character was the high and low value target (cone or rectangle) and test order (low-value or high-value test trials appear first). Each target always appeared in the same alley when they were present in the scene. The familiarization events were interleaved, so infants saw one of each event type back-to-back.

Preregistered secondary analyses used similar models and nested comparisons to test for the impact of additional factors and their interactions with trial type and trial number on infants’ log-transformed looking time. These analyses were conducted separately for habituation status (habituated vs. not habituated) and familiarization event order (low-value vs. high-value helped first). To set the habituation criteria, we calculated infants’ mean looking time to both the first three and last three familiarization events. If infants’ mean looking time to the last three familiarization events decreased by 50% or more, compared to the mean of the first three familiarization events, infants were categorized as having habituated to the familiarization events. Note that this status was determined after the experimental session and all participants saw all nine familiarization events. See *SI Appendix* for the results of these analyses.

## Supplementary Material

Appendix 01 (PDF)

## Data Availability

R Scripts; Study Materials; Preregistration Documents; and Anonymized data have been deposited in OSF (https://osf.io/zw2sy/) (47).
