# Supplementary material for: Infants use helping to infer the existence and strength of caring relationships
Source: Proc Natl Acad Sci U S A. 2026 Apr 10;123(15):e2531771123. doi: 10.1073/pnas.2531771123 (PMC13079927; doi:10.1073/pnas.2531771123)
Supplement: Supplementary file 1 — Appendix 01 (PDF) [file pnas.2531771123.sapp.pdf]

PNAS

Supporting Information for

Infants use helping to infer the existence and strength of caring relationships

*Bill Pepe[1], Brandon M. Woo[2], Ashley J. Thomas[3], & Lindsey J. Powell[1]
[1]Department of Psychology, University of California, San Diego, 9500 Gilman Dr., La Jolla, CA 92093
[2]Department of Psychological & Brain Sciences, University of California, Santa Barbara, Santa Barbara, CA
[3]Department of Psychology, Harvard University, Cambridge, MA
*Corresponding author: **Email:** wpepe@ucsd.edu

This PDF file includes:

Supporting Information text

Supporting Information Text

Methods

Additional General Methods. Families were required to have a laptop or desktop computer with a monitor; screens used for participation ranged from 10 inches to 27 inches (modal screen size was 13 inches). When participants were seated in their caregiver's lap, caregivers were instructed to close their eyes and keep them closed during the duration of the study. When participants sat in a highchair, parents sat to the side of the infant and were instructed to avoid redirecting the infant's attention towards or away from the screen.

In Experiment 1, intercoder reliability was high, as determined by intraclass coder coefficient (ICC) = .98, $p<.001$, 95% CI [0.97, 0.99]. The raw percent agreement between the two coders was 97.6%. In Experiment 2, intercoder reliability was high, as determined by intraclass coder coefficient (ICC) = .99, $p<.001$, 95% CI [0.98, 0.99]. The raw percent agreement between the two coders was 98.11%. In Experiment 3, intercoder reliability was high, as determined by intraclass coder coefficient (ICC) = .99, $p<.001$, 95% CI [0.99, 0.99]. The raw percent agreement between the two coders was 97.85%. In Experiment 4, intercoder reliability was high, as determined by intraclass coder coefficient (ICC) = .99, $p<.001$, 95% CI [0.99, 0.99]. The raw percent agreement between the two coders was 96.53%.

Experiment 1 & 2 Stimuli Details. In both the helping and hindering familiarization events, the target struggled to push a boulder up a hill. In the helping events, the boulder rolled into the scene from offscreen, the target struggled to independently move the boulder up the hill, and the helper pushed the target and boulder to the top of the hill. In the hindering events, the hinderer pushed the boulder into the target's path up the hill, the target struggled to move the boulder independently up the hill, and then the hinderer pushed the target and boulder away from

the hill. The helper or hinderer then returned to their original location, after which, the event froze until one of the two looking thresholds was met.

The goal familiarization event introduced a new scene and the target's new goal: to jump on a platform located at the end of a path between two low walls. The helper and hinderer were at the far, opposite ends of the low walls, facing the target, and appeared to track the target's movement through the path. Upon reaching the platform, the target celebrated. This event repeated three times, with a .3-second black screen appearing before the event reset each time.

Finally came alternating test events, which depicted either the helper or hinderer helping the target. The start of each event was similar to the goal familiarization, except there was now a large wall in the path that led to the platform, blocking the target's access to their goal (40). The target attempted to move towards the goal platform but was incapable of bypassing the wall. The helper and hinderer turned and tracked the target during this sequence and then one of the two pushed the wall out of the target's path and returned to their starting location. The target then proceeded through the path and celebrated reaching the platform. The trial then froze until one of the two looking thresholds was met.

Experiment 3 Stimuli Details. The setting for the goal familiarization and test trials of Experiment 3 was similar to Experiment 1 & 2, except there were two platforms behind the low wall, one to the left and one to the right. The original target from the helping and hindering familiarization trials and a novel target were positioned in the front left and right of the display, respectively. A section of the low wall extended across the middle of the display, and the helper was positioned on that middle section. The hinderer did not appear in these scenes.

The goal familiarization event consisted of a sequence of four scenes. In each scene there was a path through the low wall in front of one of the two platforms but not the other. The target

character on the same side where the path appeared went along the path to the platform, jumped on top of it, and celebrated. Then the scene reset and the path moved to the opposite side in front of the other platform. The alternative target character then performed the same set of actions. This sequence repeated twice.

In all four test trials, the low wall had paths to both platforms, but separate larger walls blocked each path. The targets simultaneously attempted but failed to bypass the walls. In alternating test events, the helper helped either the original or novel target by moving the wall out of their path and then returned to their starting location. The helper watched as the target reached the platform and celebrated.

Experiment 4 Stimuli Details. In each event, two multi-colored balls appeared from offscreen, and bounced past the helper onto two of the yellow shelves in adjacent alleys. The helper then moved forward and stopped at the ledge, equidistant from the two alleys that contained a ball. The targets then approached the shelves, attempted, but failed, to reach the balls and then returned to their starting locations. The helper then looked down into each alley that contained a ball and chose which target to help by dropping down onto the shelf in that target's alley and pushing the ball forward so both the helper and ball dropped into the bottom of the alley. The helper then rolled the ball forward towards the target of their help, who then celebrated. Each familiarization event and test trial included this identical setting and action sequence but differed based on who the helper chose to help when a different subset of targets appeared.

The familiarization events were presented in one of two orders that involved either gradually increasing or decreasing the value of the helped target (either (1) low>null, (2) medium>low, (3) high>medium, or (1) high>medium, (2) medium>low, (3) low>null). This sequence repeated three times so that each infant saw nine total familiarization events. The

cylinder-shaped character, who appeared in the middle alley, was always the target the helper valued intermediately, and the helper was always a gray, cube-shaped character.

Results

Additional Within-Experiment Results. For Experiments 1, 2, and 3, we compared looking in the helping and the hindering familiarization events. These analyses served two purposes. First, for Experiments 1 and 2, these analyses enabled us to address whether infants' looking in test trials reflected infants' expectations or instead interest in the helper and the hinderer. If looking at test reflected interest in a particular agent, and not expectations of helping, then infants should look at the same agent in the familiarization events. Second, across experiments, these analyses enabled us to determine whether infants took different amounts of time to process helping and hindering actions.

Experiment 1. We used paired sample t-tests to compare looking to helping and hindering familiarization events and found no evidence of a reliable difference in looking times: $t(51) = 0.75$, $p = .45$. For the test trials, nested model comparisons revealed that, in addition to the effect of trial type discussed in the main text, there was a significant effect of trial number on infants' log-transformed looking times, $X^2=3.99$, $p<.05$. This reflected a decrease in looking to later test trials. The interaction between trial type and trial number was not significant, $X^2=0.009$, $p=.92$.

Experiment 2. We used paired sample t-tests to compare looking to helping and hindering familiarization events and found no evidence of a reliable difference in looking times: $t(51) = 0.54$, $p = .58$. As in Experiment 1, there was a significant effect of trial number on infants' log-transformed looking times to the test trials, with looking decreasing across trials, $X^2=13.06$, $p<.001$. The interaction between trial type and trial number was not significant, $X^2=0.01$, $p=.92$.

Experiment 3. We used paired sample t-tests to compare looking to helping and hindering familiarization events and found no evidence of a reliable difference in looking times: $t(51) = 0.10$, $p = .91$. As in Experiments 1 and 2, there was a significant effect of trial number on infants' log-transformed looking times to test trials, with looking decreasing across trials, $X^2=20.35$, $p<.001$. The interaction between trial type and trial number was not significant, $X^2=0.22$, $p=.64$.

Experiment 4. We compared looking in the low-value and high-value target familiarization events to determine whether infants' looking in test trials reflected interest in a particular agent being helped. We used paired sample t-tests to compare looking to the low-value target and high-value target familiarization events and found no evidence of a reliable difference in looking times: $t(51) = 0.54$, $p = .58$. In contrast to the previous experiments, there was no effect of trial number on infants' log-transformed test trial looking times, $X^2=1.58$, $p=.20$. This is likely because test trials in this experiment were quite similar to the familiarization trials, so infants had already largely habituated to elements of the scene unrelated to the difference between trial types. The interaction between trial type and trial number was also not significant, $X^2=0.0003$, $p=.98$.

We also conducted preregistered secondary analyses to test whether infants' looking time was affected by the order of the familiarization events (high- or low-value target helped first) or habituation status. Nested comparisons revealed no simple effect of familiarization order, $X^2=0.17$, $p=.68$, nor an interaction between this factor and trial type, $X^2=0.55$, $p=.45$, or trial number $X^2=0.09$, $p=.76$. This indicates that infants did not simply expect the helper to aid whichever target it had helped most recently in the final familiarization trial.

Infants were categorized as having habituated if their mean looking time to the last three familiarization events decreased by 50% or more, compared to the mean of the first three familiarization events. According to this definition, 23 infants habituated over the course of

familiarization and 29 did not. Nested comparisons revealed no simple effect of habituation status, X^2=0.16, p=.68, nor an interaction between this factor and trial type, X^2=0.74, p=.39, or trial number, X^2=2.52, p=.11.

Comparison of Experiments 1 & 2. We also conducted preregistered analyses to test whether infants' looking time was affected by experiment (Exp 1 vs Exp 2). A main effect of experiment would suggest that infants were more interested in looking at the test trials in one of the two experiments for reasons unrelated to our hypotheses; specifically, this may have suggested infants were generally more interested in the events that included a character that did not appear in the initial helping and hindering familiarization events (i.e., the novel target character in Exp 2). Nested comparisons revealed no simple effect of experiment, X^2=0.86, p=.35, providing no evidence that infants were more or less interested in the test stimuli in Experiment 1; Original Target (raw *M*=19.0s, SD=8.91s), compared to Experiment 2; Novel Target (raw *M*=19.5s, SD=8.90s), for reasons unrelated to our hypotheses.

Comparison of Experiments 2 & 3. Prior to conducting either Experiment 2 or Experiment 3, we thought that there may be individual differences in infants' tendency to attribute relationship or disposition motives to helpers. In that case, some infants could show longer looking in Experiment 2 when a hinderer, rather than a helper, helped even a new social target, reflecting disposition-based expectations, while other infants could show longer looking in Experiment 3 when a helper switched from helping a previous target to a new one, reflecting relationship-based expectations. In this case, if the same infants participated in both Experiments 2 and 3, then their increase in looking to the "unexpected" trials relative to the "expected" trials ought to be negatively correlated across the experiments: infants whose expectations are driven by disposition inferences should show increased looking to unexpected trials in the Experiment 2 but not Experiment 3,

while infants whose expectations are driven by relationship inferences should show increase looking to unexpected trials in Experiment 3 but not Experiment 2. To test for this possibility, when we recruited participants for Experiment 2 we asked families to also sign up for a second session, within 30 days of the first, so that the infant could also participate in Experiment 3. We were able to successfully recruit 49 of 52 participants in Experiment 2 to also complete Experiment 3, providing robust power to detect a medium-sized correlation (over 80% power to detect $r < -.4$; ~70% power to detect $r < -.3$).

A correlation analysis of the difference scores between infants' looking time to the unexpected versus expected test trials in Experiments 2 and 3 revealed no evidence of a significant negative correlation across the two experiments, $r(47)=0.12$, $p=0.37$ [-0.15, 0.40], with a positive, though non-significant, observed correlation value instead. This test thus provided no evidence of reliable individual differences in infants' attribution of relationship vs dispositional motivations. Although our sample was underpowered to detect small correlations, there are other reasons to doubt the presence of such individual differences. One reason is that there was no evidence that a substantial subgroup looked longer to the "unexpected" trials in either experiment. The overall effect sizes for each were $d = 0.001$ and $d = 0.05$, and Bayes Factor analyses found moderate to strong evidence in favor of the null hypothesis of equivalent looking to the two trial types in each experiment, $BF_{01} = 12.01$(Exp2) & $BF_{01} = 7.5$ (Exp3). To compute these values, we conducted Bayesian one-sample t-tests on infants' proportional difference scores between the two types of test trials in each experiment (e.g., looking to the original target being helped – looking to the novel target being helped/total looking, in Experiment 3). Based on our data from the test trials of Experiment 1, we defined the alternative hypothesis with an effect size of $d = 0.45$ and a scale of .707. Thus, we think the best explanation of the data across all experiments is the one offered in

the main text: that infants in general appealed to relationships rather than dispositions as the cause of actors' social behavior, and that they were able to learn about the relative strength of an actor's multiple relationships. This supported expectations about the future behavior of helpers and hinderers when directed toward previously observed targets (Experiments 1 & 4) but failed to support expectations toward a novel target (Experiment 2). In Experiment 3, infants' equal interest in the two test trial types, which involved the helper prioritizing either a previous target or a novel target, is potentially explained by infants' ability to use such events to extract new information about relative strength of care, no matter who a helper chose to assist.
